# The Future in Craniofacial Surgery: Computer-Assisted Planning

**DOI:** 10.5041/RMMJ.10079

**Published:** 2012-04-30

**Authors:** Stephen A. Schendel, Hagai Hazan-Molina, Adi Rachmiel, Dror Aizenbud

**Affiliations:** 1 Craniofacial Anomalies Center, Packard Children’s Hospital, Stanford University School of Medicine, Palo Alto, CA, USA;; 2 the Orthodontic and Craniofacial Department, School of Graduate Dentistry Rambam Health Care Campus, Bruce Rappaport Faculty of Medicine, Technion-Israel Institute of Technology, Haifa, Israel; and; 3 the Oral and Maxillofacial Surgery Department, Rambam Health Care Campus, Bruce Rappaport Faculty of Medicine, Technion-Israel Institute of Technology, Haifa, Israel

**Keywords:** Computer-Assisted, Craniofacial, Imaging, Three-Dimensional, Surgery

## Abstract

Advancements in computers, prototyping, and imaging, especially over the last 10 years, have permitted the adoption of three-dimensional imaging protocols in the health care field. In this article, the authors present an integrated simulation system for craniofacial surgical planning and treatment. Image fusion technology, which involves combining different imaging modalities, was utilized to create a realistic prototype and virtual image that can be manipulated in real time. The resultant data can then be shared over the Internet with distantly located practitioners.

## THE NEW, NEW WORLD OF BIOTECHNOLOGY

Significant advances in biotechnology, biosensors, and medical informatics have occurred mainly within the past decade, leading to improved prototype production, imaging, and simulation in the health care field.^1^,^2^ As the pace of innovation increases, even more biomedical applications will be developed. The extrapolation of these current technological trends into the future is based on the fact that these systems are all web-based and therefore do not encounter communication barriers. In addition, the advanced computational technology and the unrestricted sensing devices, which are unnoticeable, leave the limits unbounded.^1^,^2^

Advanced technologies comprising microprocessors have become more powerful, cheaper, and consume less energy.^1^,^2^ Sensing technologies have become highly specific, microminiaturized, and even implantable. Multiple ubiquitous wireless infrastructures now exist for cellular phones, WiFi, and WiMax network accounts, enabling integration of information to become the norm.

**Figure 1:**
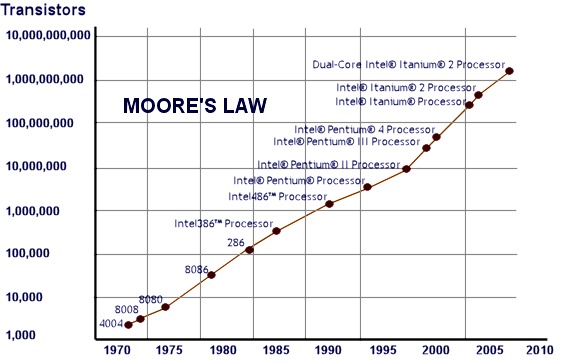
**Moore’s law diagram—suited to 2010.**

Human anatomy models, produced with different technologies combining images captured in the digital imaging and communications in medicine (DICOM) format, are processed using specific three-dimensional reconstruction software. This software has a minimum material deposition thickness to form a build layer. The thinner this layer, the better the surface finishing, and the smoother the prototype surface.^3^ In 1965, Gordon Moore sketched his prediction of the pace of silicon technology.^4^ Decades later, Moore’s law (Figure 1) has remained true, as the number of transistors on a chip roughly doubles every two years. Consequently the scale continues to become smaller, while transistor counts climb. Along the same trend the ability to increase device complexity and integrate many capabilities onto one chip is growing. The cumulative impact of these spiraling advancements in capabilities empower the economy and the Internet, running on everything from digital phones and PCs to stock markets, spacecraft, and medical devices, facilitating today’s information-rich, converged digital world.

## KEY APPLICATION AREAS FOR CRANIOFACIAL SURGERY

### Anatomical Databases: Data for Simulation and Planning

Three-dimensional (3D) anatomic relationships are difficult to learn. Advanced visualization techniques can help people learn better. The use of advanced imaging modalities such as computerized tomography (CT), surface imaging, serial section, and synchrotron can improve visualization and lead to a better understanding of anatomical data and structural relationships.

With the development of information technology, 3D models can be devised and built, based on virtual prototypes by means of a computer numerical control (CNC) device. Computers can now be used to create accurately detailed projects that can be assessed from different perspectives in a process known as computer-aided design (CAD). To materialize virtual objects using CAD, a computer-aided manufacturing (CAM) process has been developed. To transform a virtual file into a real object, CAM operates using a machine connected to a computer, similar to a printer or peripheral device.^5^

**Figure 2:**
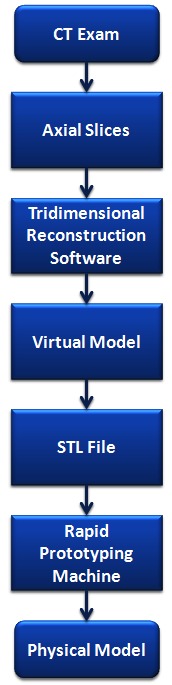
**Step-by-step sequence to fabricate a prototype of a human anatomic skull.** Attention: To fabricate the prototype, images should be stored in DICOM format and recorded in a CD-ROM or sent via the Internet to the prototyping bureau; the CT scan films are not necessary.^3^

An actual 3D model can be built to reproduce an anatomy of a patient based on CT images obtained during that patient’s examination, thanks to advances in CT scanner quality and the development of specific software for this purpose.^6^ Manufactured according to CT, images are not exactly prototypes, but rather replicas, because they are not created by a designer or planner, but replicated (Figure 2).^3^

**Figure 3:**
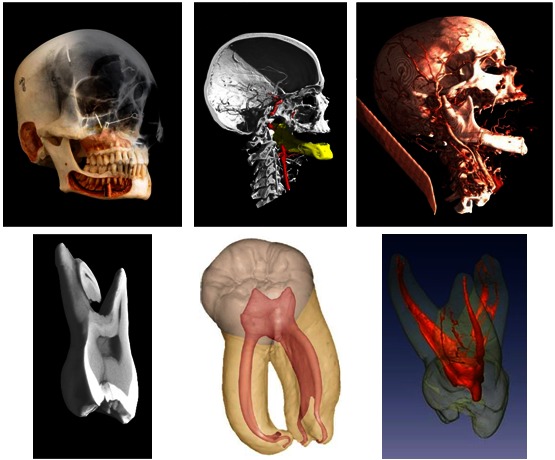
**Use of advanced imaging provides a comprehensive atlas of craniofacial and dental anatomy.**

The new imaging technology provides actual models and comprehensive atlases of every part of the human body in all the anatomical variations, pathologies, and developmental and evolutionary complexities (Figure 3).

**Figure 4:**
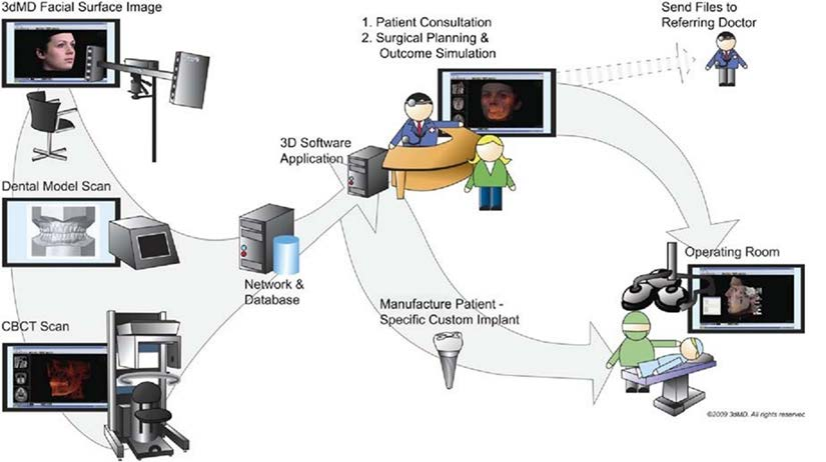
**Work flow diagram for the creation and distribution of patient-specific data and treatment planning.** Reprinted from reference 1, with permission from the American Association of Oral and Maxillofacial Surgeons.

The benefits of computers assisting surgeons in the operating rooms include intraoperative planning according to the available advanced data. Modification of the preliminary treatment plans and simulation is possible as well.^1^ The typical work flow scenario can be seen in Figure 4. The initial data are acquired from multiple sources such as a cone beam CT (CBCT) scan, facial surface image, and dental model scans. These are then transferred to the network for database storage and file manipulation. The system then produces the patient-specific anatomic reconstruction (PSAR), and the treatment planning is completed.^1^,^2^ All treating physicians can thus be directly involved in the creation of the plan. Finally, the custom implants and splints can be created, and data are sent directly to the surgical team for surgical guidance and assistance.

### Patient-Specific Computer-Based Surgical Planning

**Figure 5:**
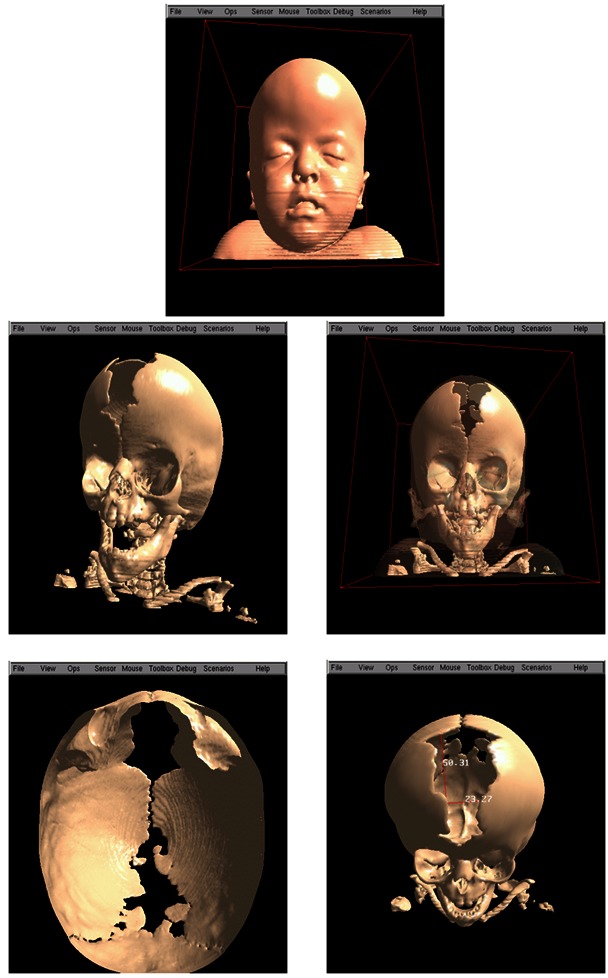
**Craniosynostosis of a 1-year-old female virtual patient created by the PSAR, to develop and simulate treatment protocols.**

The adoption of 3D imaging protocols and the power of the Internet are advancing diagnosis, treatment planning, and outcome evaluation toward the next-generation paradigm. They enable the creation of an accurate prototype and electronic patient in the real world, which magnifies the potential for truly patient-centered care.^1^,^2^,^7^ The actual patient model created by CAD-CAM systems and the virtual patient created by the PSAR can then be studied and used for surgical training and to develop simulated treatment protocols (Figure 5).^1^,^2^

The ability preoperatively to plan a surgical procedure and evaluate outcomes can provide a better surgical result, potentially in less time and with fewer expenses incurred in the operating room, and less surgical revision will be required. In this way predictable results improve, with increased surgical precision and lower surgical risks and comorbidity. Operating time decreases, while its efficiency increases. The overall upgrading leads to lower surgical costs.^1^,^2^,^7^

### Craniofacial Surgical Planning Process

Once the raw data are viewed interactively, the system also supports the automated segmentation of these data to generate a 3D geometric computer model.^1^,^2^,^8^ At this stage, since the mesh has been generated in the same world space as the original voxel data, we can provide an integrated, registered geometric and volumetric display for the user to verify and understand the patient’s condition.^1^,^2^

**Figure 6:**
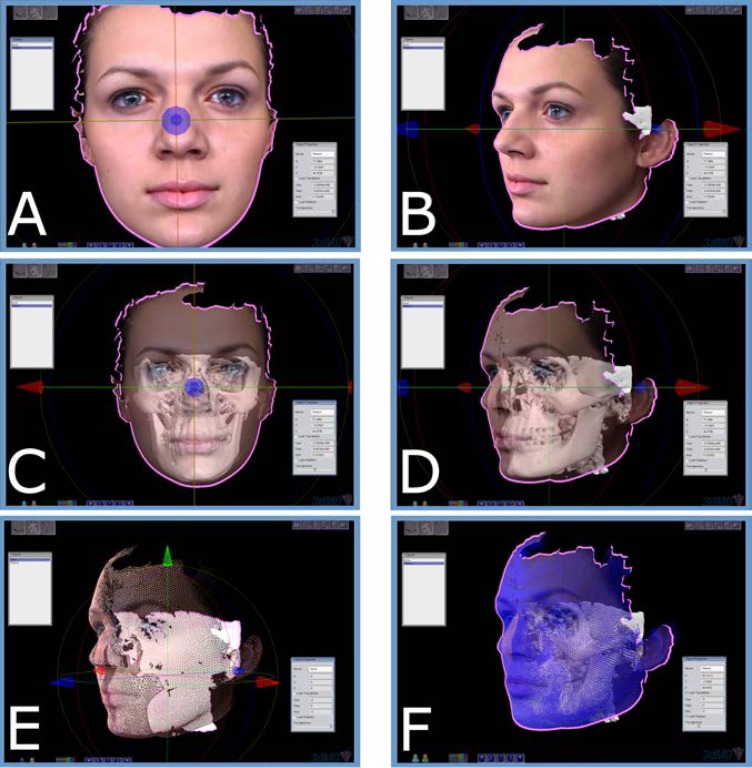
**Series of interactive tools for 3D analysis are provided to quantify the patient’s condition.^2^** **A:** Surface scan image overlaid on generated skin mesh. Frontal view. **B:** Lateral views. **C:** Patient with skin surface semi-transparent to reveal underlying bone model. Frontal view. **D:** Lateral view. **E:** Wire mesh view of skin and underlying bone model. **F:** Wire mesh view of skin and bone model displaying underlying tie-nodes to model soft-tissue compressibility. These nodes act as springs that are differentially calibrated to represent differing tissue compressibility from the bone polygons to the nodes. Thus, this determines how the skin and bone interact.

A series of interactive tools for 3D cephalometric analysis are provided for measuring distances and angles and identifying landmarks to quantify the patient’s condition. In the virtual environment, the patient can be rotated and examined from multiple views in real time with simple movement of the mouse, or multiple views can be simultaneously viewed on a divided screen (Figure 6).^1^,^2^

### Interaction and Simulation

The previous steps provide the basis for visualization and examination of the patient’s current condition; advancing toward prediction of surgical outcome requires the use of simulation.^9^

**Figure 7:**
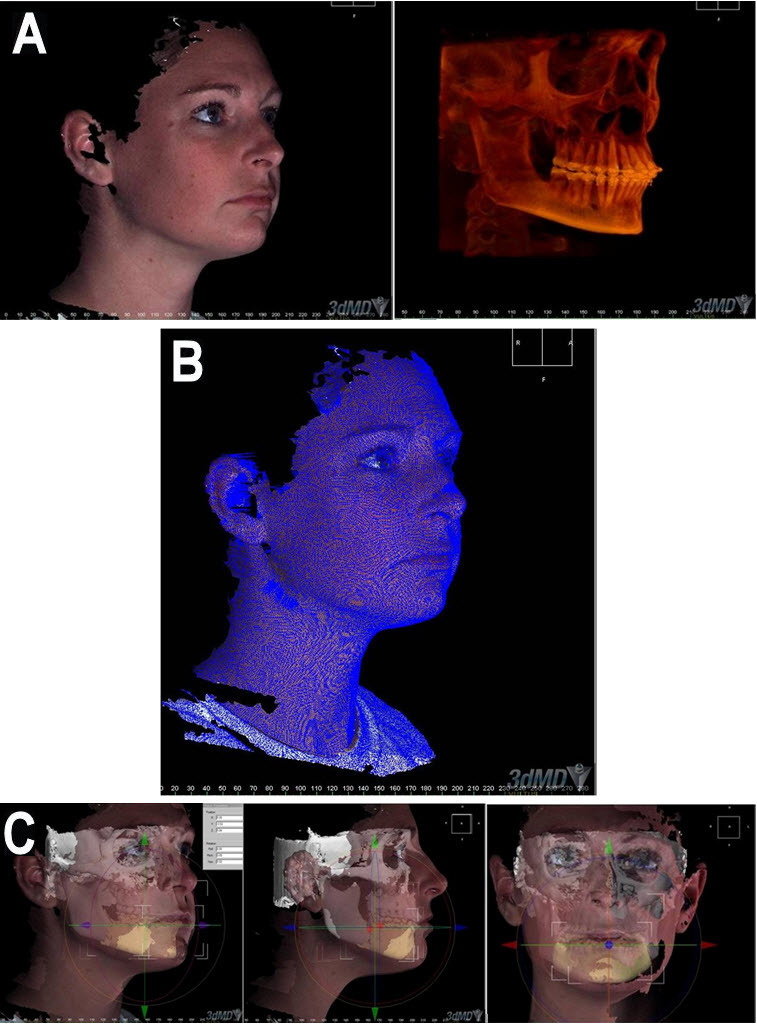
**Computer-based simulation.** A simulation gives the clinician the opportunity to perform virtual surgery and to predict the surgical outcome, thereby increasing the probability of successful results with no risk to the patient. **A:** Presurgical orthognathic case imaging. **B:** Applying the mass-spring model. **C:** Surgical simulation—mass-spring approach.

Simulation refers to an imitation of a real-world process in a computer program using mathematic models to study the effects of changing the parameters and conditions in order to make a decision.^9^ Computer-based simulations give the clinician the opportunity to perform virtual surgery or treatment while increasing the probability of a successful outcome, with no risk to the patient. This allows an alternative approach.^1^,^2^,^9^ The mass-spring model technique of simulation involves implementing a biomechanical model that defines the relationship between the hard and soft tissue with hundreds of thousands of non-linear connector points (Figure 7).^9^ This generates 3D deformable tissue models that include spring-based force computations to model the physical characteristics of real tissue reactions. The models use force computations from physical laws and apply these forces to the 3D model components. The computations modeled include tissue deformation and relaxation, external forces such as gravity, and 3D collision detection with force feedback. This type of interaction moves the world of simulation to a practical basis, from the computing laboratory to the clinic’s desktop computer.^1^,^2^,^9^

The geometric model of the patient’s bone and soft-tissue structure produced in previous steps is used with a mass-spring engine to model the soft-tissue dynamics.

The system currently supports both rigid-body kinematic simulations appropriate for modeling the bone, as well as mass-spring simulations of soft tissues. Thus, the patient’s bone can be represented in the system as having the dynamics of a rigid object, whereas the skin surface can be modeled using soft-tissue simulation (Figure 7).^1^,^2^,^9^

**Figure 8:**
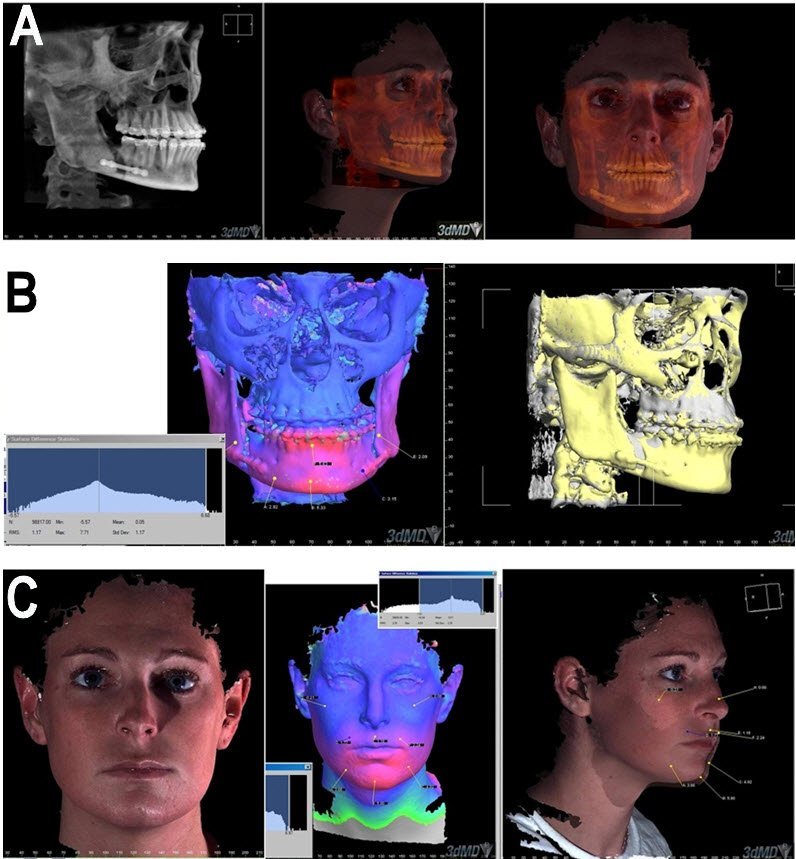
**The same patient depicted in Figure 6, post-surgery imaging.** **A:** Patient’s specific anatomical reconstruction with CBCT imaging. **B:** Actual bone change from treatment. **C:** Actual soft-tissue change from treatment.

A number of virtual instruments and abstract interaction objects have been implemented to allow the user to perform osteotomies and other operations on the patient model.^1^,^2^,^9^ Using these tools, the user can cut bone and reposition in real time since the simulation system recalculates the soft tissues of the skin on top of the new bone structure. Refined cutting tools permit anatomically correct osteotomies of the maxilla or mandible. Specific measurements of parameters such as surgical movement can be calculated. The virtual simulation can then be used to preplan surgical procedures such as the shape and size of fixation plates or other implants. In addition, the placement of the virtual model on the web permits many individuals in different locations to view and discuss the case and treatment plan (Figure 8).^1^,^2^,^9^

## CONCLUSIONS

Advancements in computer imaging have revolutionized the treatment of dentofacial deformities and, specifically, orthognathic surgery. Prototyping, computer imaging, and simulation can provide significant benefits for both the professional and the patient. Greater precision and accuracy in diagnosis and surgery can be obtained by means of virtual training. The surgeon’s performance can improve using these systems for training, and it is risk-free. All of this increases the patient’s safety and improves the outcome. Recent technical advances have made computer imaging more realistic and user friendly and have lowered the cost. The ability to make these systems web-based adds another facet by increasing availability. Team members, even though they may be distant from one another, can simultaneously evaluate treatment options in real time. The continuous changes in this field will be associated with the ever-increasing adoption of computer imaging and simulation in medicine and surgery, forever changing the practice of medicine.

## Abbreviations:

3D: three dimensions/three-dimensional;
CBCT: cone beam CT;
CT: computerized tomography;
PAC: picture archiving and communication;
PSAR: patient-specific anatomic reconstruction.
